# A High-Sensitivity Current Sensor Utilizing CrNi Wire and Microfiber Coils

**DOI:** 10.3390/s140508423

**Published:** 2014-05-12

**Authors:** Xiaodong Xie, Jie Li, Li-Peng Sun, Xiang Shen, Long Jin, Bai-ou Guan

**Affiliations:** Institute of Photonics Technology, Jinan University, Guangzhou 510632, China; E-Mails: xxd7288100@gmail.com (X.X.); 741_okm@163.com (L.-P.S.); xgsx1989@163.com (X.S.); iptjinlong@gmail.com (L.J.); tguanbo@jnu.edu.cn (B.G.)

**Keywords:** microfiber, current sensor, sensitivity, chrome-nickel wire

## Abstract

We obtain an extremely high current sensitivity by wrapping a section of microfiber on a thin-diameter chromium-nickel wire. Our detected current sensitivity is as high as 220.65 nm/A^2^ for a structure length of only 35 μm. Such sensitivity is two orders of magnitude higher than the counterparts reported in the literature. Analysis shows that a higher resistivity or/and a thinner diameter of the metal wire may produce higher sensitivity. The effects of varying the structure parameters on sensitivity are discussed. The presented structure has potential for low-current sensing or highly electrically-tunable filtering applications.

## 1. Introduction

Optical fiber current sensors have been extensively studied due to their characteristics such as immunity to electromagnetic interference, inherent safety, and low weight, compared with traditional electric sensors. Previously, many fiber current sensors have been realized by the use of magneto-optics interaction effects, such as Faraday rotation or magnetostrictive effects. Nevertheless, the Verdet constant of silica fiber is very low and thereby the formed fiber architecture is normally huge [1]. Doping the silica fiber with some rare elements such as terbium or europium can enhance the magneto-optics efficiency of the structure to a great extent and thus the electric-current responsivity may be improved, but the manufacturing cost increases and the temperature cross-sensitivity may be enhanced as well. Another selectable method is to coat a fiber Bragg grating section with a thin conductive metallic material [2]. The current flowing in the coating on the surface of fiber can induce a temperature variation and hence shift the resonant Bragg wavelength. However the fabrication requires the use of the state-of-the-art coating equipment.

Recently, microfiber devices with sub-wavelength scales have attracted great interest because of their compactness, high flexibility, and large evanescent field effect [3,4]. Using the self-coupling effect of the adjacent microfibers, a number of resonators with microfiber loops [5], knots [6], and coils [7,8] have been realized, which may produce very low transmission losses and high Q values [7,8]. It has been found that when a copper rod is surrounded with a microfiber loop resonator, the dip wavelength can be shifted by the applying current [9]. This finding opens a possibility of electric current sensing utilizing the temperature variation in a metal-microfiber structure. The sensitivity is defined as the wavelength shift per unit change of the square of current. So far the realized sensitivity is 0.0265–7.5 nm/A^2^ within a typical current range of 0–2 A [9–13]. In this work, a very high sensitivity is achieved with assistance of a thin chromium-nickel (CrNi) wire and a microfiber coil resonator. The measured sensitivity is as high as 220.65 nm/A^2^, which is two orders of magnitude higher than other metal-microfiber configurations described in the literature, thus exhibiting great potential for high-sensitivity current sensing or electrically tunable filtering applications.

## 2. Experimental Setup

**Figure 1.**
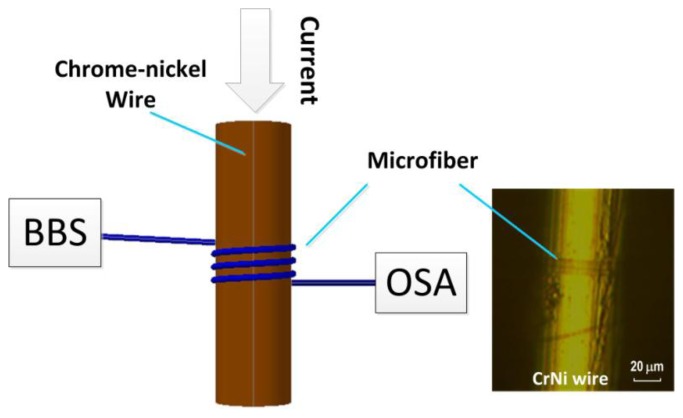
Schematic of our electric current sensor with a microfiber and a CrNi wire. Inset shows a micrograph of a fabricated structure.

Figure 1 shows the configuration of our electric current meter, which contains a subwavelength-scale microfiber spirally wrapped around a non-magnetic chromium-nickel (CrNi) wire. The microfiber is obtained by locally heating and stretching a standard single-mode fiber with assistance of the conventional flame-brushing technique [4]. The fiber diameter is tapered to 2–6 μm by controlling the fiber stretching speed. Like the well-established biconical model [4], the tapered fiber comprises two transition regions on both ends and a minimum waist in the center. We subsequently wrap the uniform waist onto the CrNi wire that contains 20% chromium and 80% nickel. To do so, we mount the CrNi wire onto a precision rotational stage as described in [8]. The microfiber may be wrapped around the metal wire when one fiber end is fixed on the rotational stage and another one end is pulled straight. In our experiments the spacing between adjacent microfiber turns can be changed slightly by tuning the pulling force. The spacing of two adjacent microfibers turns is no more than 4 μm, which can produce strong mode coupling of the microfibers. The adopted CrNi-wire diameters are 50 μm, 60 μm, and 80 μm, respectively. Thanks to the strong van der Waals and the electrostatic attraction forces between the microfiber and the metal wire, the device is relatively stable and robust.

**Figure 2.**
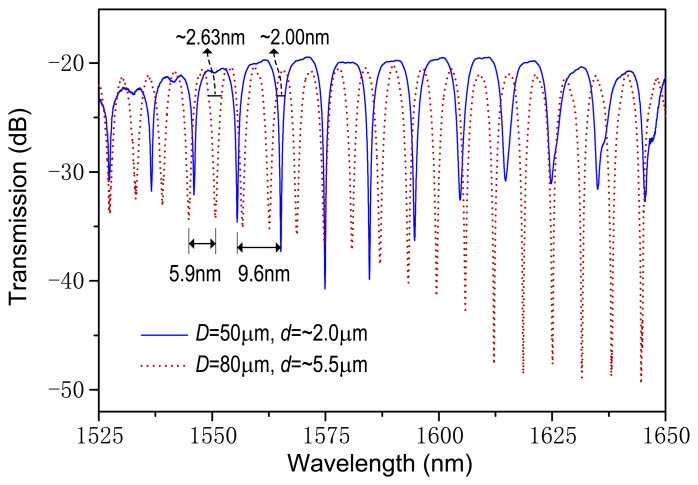
Transmission spectra of our device with different sizes of the CrNi wire and the microfiber.

Figure 2 records the transmission spectra for the metal-wire diameters of *D* = 50 μm and 80 μm and the microfiber diameters of *d* = ∼2.0 μm and ∼5.5 μm, respectively, measured using a broad-band light source (BBS) and an optical spectrum analyzer (OSA). As shown in Figure 1, the input mode field experiences self-coupling between microfibers through the evanescent mode field and thus the resonant spectrum occurs at the microfiber output. The free spectral range (FSR) is mostly dependent on the diameter of the CrNi wire. A larger value of *D* may generate a wider FSR. From Figure 2, the measured FSRs are about 9.60 nm and 5.90 nm and the estimated full-widths at half-maximum are ∼2.00 nm and ∼2.63 nm, corresponding to the thinner and thicker metal wires, respectively. Note that the spectral characteristics can be optimized by tuning the number of microfiber coils around the metal wire. In our experiment, the coil number used is four, which gives a compact device length of less than 35 μm. The extinction ratio of spectrum can be higher than 20 dB from Figure 2. The transmission loss is around 20 dB, which is acceptable for electric current measurements. Our investigation also shows that for a microfiber diameter of ∼5 μm, each loop of microfiber coil may produce a loss increment of around 5.0–7.0 dB, mainly due to the absorption of the metal material.

## 3. Results and Discussion

**Figure 3.**
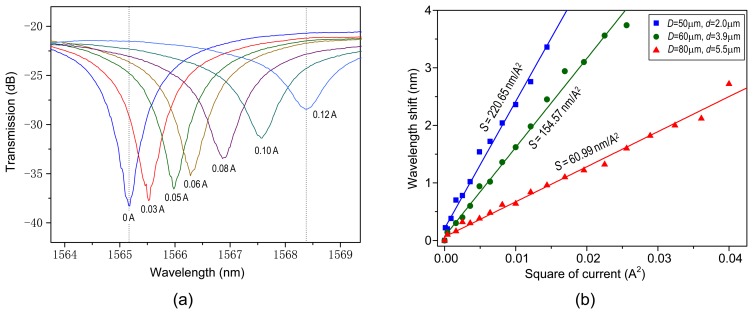
(**a**) Variation of transmission spectrum with the change of the carried current. The resonant wavelength shifts from 1,565.2 nm to 1,568.4 nm when the current varies from 0 A to 0.12 A; (**b**) Dip wavelengths as functions of the square of current for the different component sizes. The points are experimental values and the solid lines are linear fitting results.

Figure 3a illustrates the transmission spectra at different electric currents, for the parameters *D* = 50 μm and *d* = 2.0 μm at the room temperature. The dip wavelength redshifts from 1,565.2 nm to 1,568.4 nm when the applied current increases from 0 to 0.12 A, which produces a sensitivity of around 220.65 nm/A^2^. Figure 3b details the dip wavelengths as a function of the square of current, for the parameters *D*/*d* = 50 μm/∼2.0 μm, 60 μm/∼3.9 μm, and 80 μm/∼5.5 μm, respectively, around 1,565 nm. The points are the experimental data and the solid lines are the linear fitting results.

We can see that the wavelengths increase almost linearly with an increase in the square of current. The wavelengths are measured with a current increment of 0.01 A at each step. As the current varies, the dip wavelength shifts promptly and becomes steady within about one minute. From Figure 3b, our measured sensitivities are 220.65 nm/A^2^, 154.57 nm/A^2^, and 60.99 nm/A^2^, respectively. In fact, the smaller *D* or *d* may be ready to generate the larger sensitivity, as detailed later. As shown in Figure 3b, the maximum currents are 0.12 A, 0.16 A, and 0.20 A, respectively. A too large current should be avoided because a very high temperature may soften the silica microfiber and thus induce an irreversible variation in the spectrum. In a proper current range, our structure exhibits good repeatability and reconfigurability, making it appropriate for low-current sensing or electrically tunable filtering. Table 1 indicates electric-current responsivities of the metal-microfiber structures recently reported in the literature. The measured sensitivity has been improved by over two orders of magnitude compared to those other configurations. The precision is defined as the minimum current variation that can be detected in the OSA. Considering a wavelength resolution of OSA, δλ, the precision can be expressed as δ*I* = δλ/(dλ/d*I*) = δλ/(2*I*·*S*). It is obvious that the precision is mainly determined by the sensitivity and the resolution of OSA. Giving *S* = 220.65 nm/A^2^ and δλ = 0.06 nm for our OSA, we obtain δ*I* = 1.36 mA at 100 mA.

**Figure 4.**
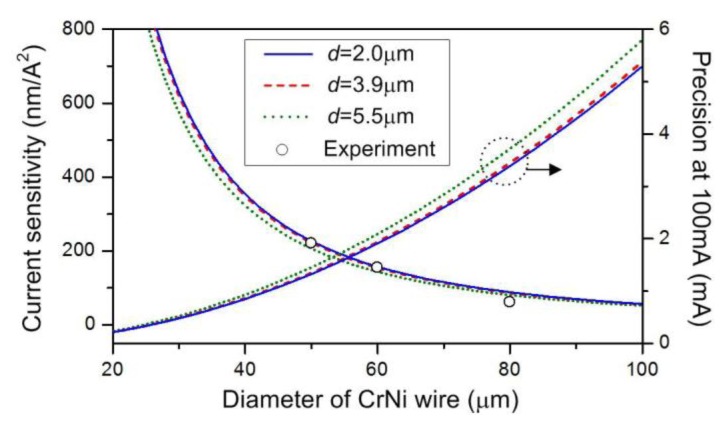
Current sensitivities as functions of the CrNi diameter, corresponding to the microfiber sizes of *d* = 2.0, 3.9, and 5.5 μm, respectively. The curves are the calculated values and the points are our experimental results. A precision at 100 mA is achieved considering a 0.06 nm resolution of the OSA.

To investigate the influence of physical parameter to the current sensitivity, we note that the dissipated power from the carrying electric current in the conduction wire can give rise to an instantaneous temperature variation Δ*T*, with the relationship of Δ*T* = (*K*ρ*h*/σ)·Δ*I*^2^ [5,6], where *K* is the constant of dissipation, Δ*I*^2^ is the change of the square of current, ρ is the resistivity of the metal wire, *h* is the structural length, and σ is the cross-sectional area of the metal wire. The temperature variation can influence the effective index *n*_eff_ and the length *L* of each microfiber coil and hence shift the transmission spectrum. In a microfiber resonator, the resonant condition is given by *n*_eff_*L* = *m*·λ_res_, with *n*_eff_ the effective index and *m* an integer. Then the wavelength shift Δλ can be expressed as:

(1) $$\Delta \lambda =\lambda N\left(\alpha +\beta \right)\Delta T=\frac{C\lambda N\rho }{D^{2}}\Delta I^{2}$$

where α and β represent the thermal expansion and thermo-optic coefficients of microfiber, respectively, *N* = *n*_eff_/[*n*_eff_ − λ (∂*n*_eff_/∂λ) ] represents the dispersion factor of the mode effective index *n*_eff_, and *C* ∝ 4 *Kh*(α + β)/π. From Equation (1), the wavelength shift Δλ is proportional to the change of the square of current Δ*I*^2^, consistent with the observation in Figure 3. The current sensitivity, as given by *S* = Δλ/Δ*I*^2^ = *C*λ*N* ρ/*D*^2^, is principally determined by the conductor resistivity ρ and the metal diameter *D*. A larger metal resistivity and/or a thinner metal wire diameter may produce a higher current sensitivity mostly due to its stronger electric-thermal conversion efficiency. In our experiment, the resistivity of the CrNi wire is around 1.09 × 10^−6^ Ω·m, which is much larger than that of the copper 1.68 ×10^−8^ Ω·m [10]. Considering the parameters *D* = 50 μm and *d* = 2.0 μm, we can obtain *n*_eff_ = 1.353 and *N* = 0.884 at λ = 1,550 nm by solving the wave equation in a silica microfiber [4]. By substituting the values to Equation (1), we have *C* ≈ 3.46 × 10^−4^ m/(Ω·A^2^). Figure 4 plots (a) the sensitivity and (b) the measurement precision, respectively, as a function of CrNi diameter for the different microfiber sizes of *d* = 2.0 μm, 3.9 μm, and 5.5 μm. The curves are the calculated results and the points are the experimental values, which match each other well. Analysis shows that the influence of microfiber diameter on sensitivity or precision is relatively small. With the decreasing metal wire size, the device sensitivity increases, leading to improvements of the measurement precision. Note that our structure is implemented only by binding microfiber onto the CrNi wire with the van der Waals and the electrostatic attraction forces. To further improve the stability of the device, one may package the combination with a low-index polymer such as Teflon. Then the device performance might be changed and related to the Teflon's optical and thermal properties [13]. The packaging polymer may however be softened by a high current and thereby the maximum current should be limited.

## 4. Conclusions

In conclusion, we report a high-sensitivity electric current sensor manufactured by wrapping a microfiber onto a thin-diameter CrNi wire. The measured current sensitivity can be as high as 220.65 nm/A^2^ for a device length of only 35 μm. Such a sensitivity is two orders of magnitude higher than other metal-microfiber configurations reported in the literature. Considering a 0.06 nm resolution in the OSA, the detectable precision is ∼1.36 mA around 100 mA. The effects of varying the component sizes on the device sensitivity are discussed. The experimental results show good agreement with the theoretical analysis. The presented device exhibits potential for low-current sensing or electrically-tunable filtering applications.

## Figures and Tables

**Table 1.** Electric-current sensitivities utilizing the thermal effect for the metal-microfiber structures.

**Microfiber Structures**	**Sensitivity**
Microfiber loop & copper wire	0.0265 nm/A [9]
Microfiber knot & copper rod	0.0513 nm/A^2^ [10]
Microfiber Mach-Zehnder interferometer & copper wire	0.54 nm/A^2^ [11]
Erbium-doped microfiber knot & copper wire	0.70 nm/A^2^ [12]
Microfiber coil & nichrome with Teflon tube	7.5 nm/A^2^ [13]
Our present device	220.65nm/A^2^
